# Surveying the floodgates: estimating protein flux into the endoplasmic reticulum lumen in *Saccharomyces cerevisiae*

**DOI:** 10.3389/fphys.2014.00444

**Published:** 2014-11-13

**Authors:** Michael Vincent, Mark Whidden, Santiago Schnell

**Affiliations:** ^1^Department of Molecular & Integrative Physiology, University of Michigan Medical SchoolAnn Arbor, MI, USA; ^2^Department of Molecular, Cellular, and Developmental Biology, University of MichiganAnn Arbor, MI, USA; ^3^Department of Computational Medicine & Bioinformatics, University of Michigan Medical SchoolAnn Arbor, MI, USA; ^4^Brehm Center for Diabetes Research, University of Michigan Medical SchoolAnn Arbor, MI, USA

**Keywords:** endoplasmic reticulum, protein flux, translocon, protein import, Sec61, unfolded protein response

## Abstract

Endoplasmic reticulum resident proteins, along with all proteins traveling through the secretory pathway must enter endoplasmic reticulum lumen through membrane-embedded translocons. In *Saccharomyces cerevisiae* the heterotrimeric endoplasmic reticulum translocon is composed of the Sec61p, Sss1p, and Sbh1p core subunits. While the involvement of various molecules associated with the Sec61 complex has been thoroughly characterized, little attention has been given to the overall flux through these channels. In this work we carried out a meta-analysis to estimate the average and absolute flux of proteins into the endoplasmic reticulum lumen. We estimate an average of 460 proteins enter the endoplasmic reticulum every second, with an absolute minimum and maximum flux of 78 and 3700 molecules per second, respectively. With current technologies limiting the ability to obtain accurate measurements of these events, our estimates shed light on the flow of protein entering the endoplasmic reticulum lumen.

## Introduction

During the past few decades the research community has gathered an immense amount of information regarding the function and processes of the endoplasmic reticulum (ER). It is now well understood that this organelle marks the start of the secretory pathway, and orchestrates the folding, modification, and assembly of approximately one third of the eukaryotic proteome. Various physiological conditions, such as increases in protein folding demand or protein flux into the ER lumen, are capable of inducing the upregulation of ER protein folding machinery [for a detailed review, see (Schroder and Kaufman, 2005)]. Understanding the dynamic nature of ER proteostasis is particularly relevant to the investigation of protein misfolding diseases, many of which are characterized in part by the accumulation of misfolded protein in the ER lumen. Recent theoretical work modeling the ER as a continuous flow reactor has identified the inflow of unfolded proteins into the ER as a critical factor for determining threshold behavior of protein misfolding (Sandefur and Schnell, 2011), and support for this prediction has been obtained experimentally (Wright et al., 2013). Although the current of nascent unfolded polypeptides flowing into the ER lumen is recognized as important to understanding protein misfolding diseases, neither theoretical nor experimental attempts have been made to quantify the number of proteins entering the ER in a given unit of time.

High protein traffic is concomitant with high flux through membrane-embedded translocons that function as the proteinaceous gateway to the lumenal space. Indeed, this traffic can vary greatly depending on both cellular demand and the protein folding capacity of the ER itself. For instance, when the accumulation of unfolded and/or misfolded protein exceeds the capacity of the ER folding machinery, the ER exhibits a state of stress. To regain proteostasis, the ER activates the unfolded protein response (UPR), an evolutionarily conserved homeostatic mechanism. In yeast, the Inositol-requiring enzyme 1 exclusively mediates UPR activation, and consequently leads to the upregulation of UPR-target genes encoding protein-folding machinery (Lee, 1987; Kozutsumi et al., 1988; Shamu and Walter, 1996; Sidrauski et al., 1996; Sidrauski and Walter, 1997). However, although much is known regarding ER proteostasis and the circumstances capable of perturbing it, the basal current of protein flowing into the ER remains largely uncharacterized from a quantitative standpoint. Lacking this fundamental knowledge, it is difficult to truly evaluate the specific effect of state-altering perturbations on the ER. Furthermore, efforts to model processes of the ER have been hindered by the absence of this information as well. Current models utilizing unfolded protein source parameters have relied on parameter fitting techniques or assumptions based on biological intuition, but have not used values based on translocation measurements (Pincus et al., 2010; Chambers et al., 2012).

Motivated by the absence of objective measurements of protein import into the ER, we carried out a systematic meta-analysis of proteomic and kinetic data relevant to ER translocation in eukaryotes. We provide a novel estimate of the total import of nascent unfolded polypeptides into the lumen. Furthermore, our method enables others to estimate the flux of any yeast protein localizing to the ER (including both ER-resident and transient proteins). To our knowledge, this work serves as the first quantitative data-driven estimate of protein flux into the ER in yeast.

## Materials and methods

### Defining the population of ER-resident and transient proteins

By analyzing TAP-tagged strains with a quantitative western blotting approach, Ghaemmaghami et al. determined the single-cell abundances of a majority of the *Saccharomyces cerevisiae* proteome (Ghaemmaghami et al., 2003). The subcellular localization of the yeast proteome has also been determined as well. This was accomplished by analyzing protein localization in cells transfected with green fluorescent protein fusion constructs prepared for all open reading frames (ORFs) predicted in yeast (Huh et al., 2003). The latter study identified 296 ORFs encoding proteins localizing to the ER (Huh et al., 2003). The abundances of 23.6% (70/296) of these ORFs were unable to be quantified experimentally (Ghaemmaghami et al., 2003). Nevertheless, together the 226 quantifiable ORFs encode 3,972,824 ER-localized proteins, and we assume this value represents the total ER protein population. With the population of proteins in place, we next set out to define the population of ER translocons that serve as the entry points for all proteins traveling into the ER.

### ER translocon abundance estimates and their corresponding kinetic parameters

Proteins destined for ER import traverse the membrane via either cotranslational translocation (signal-recognition particle-dependent; SRP-dependent) or posttranslational translocation (SRP-independent) (Katz et al., 1977; Glabe et al., 1980; Hann and Walter, 1991; Ng et al., 1996; Matlack et al., 1999). While differences in molecular machinery exist for each process, Sec61p, Sec62p, Sec63p, Sss1p, and Kar2p (the homolog of the mammalian chaperone BiP) have been identified as common translocon requirements for both processes (Deshaies and Schekman, 1987, 1989; Vogel et al., 1990; Esnault et al., 1993, 1994; Brodsky et al., 1995).

Much remains unknown regarding the specific stoichiometry of the ER translocon. The mammalian Sec61 complex is purified as a heterotrimer, leading many to believe this complex consists of equal numbers of Sec61α, Sec61β, and Sec61γ subunits (Gorlich and Rapoport, 1993). In yeast, Sec61p-Sss1p- Sbh1p represents the corresponding heterotrimer. However, while Sec61 and Sss1 have been demonstrated as essential, this is not the case for Sbh1 as deletion mutants are viable with only minor protein transportation defects (Finke et al., 1996). Thus, we used the required components (core and auxiliary) encoded by essential genes to define the minimum number of ER translocons present. Fortunately, the cellular abundance has been determined for all but one of these components (Sss1p abundance is unknown). In yeast, Sec61p, Sec62p, and Sec63p are present at 24,800, 16,500, and 17,700 molecules per cell, respectively (Ghaemmaghami et al., 2003). Kar2p is highly abundant at 337,000 molecules per cell, and can be immediately ruled out as a limiting factor for translocon assembly (Ghaemmaghami et al., 2003). Thus, assuming one molecule of each subunit is present per translocon, we arrive at an estimate of 16,500 ER translocons per cell, which matches the abundance of the limiting Sec62p subunit [inferred from proteomic information obtained from Ghaemmaghami et al. (2003)].

Kinetic parameters relevant to ER translocation are currently unavailable in yeast, however, the rate of translocation has been determined in COS-I cells (Goder et al., 2000). By monitoring the translocation of an N-terminal domain across the ER membrane, Goder et al. (2000) determined this process to occur at a rate of 8.0 ± 1.4 amino acids per second. Assuming a normal distribution, the 95% confidence interval of the translocation rate is 8.0 ± 1.1 amino acids per second. Note that the confidence interval of the average translocation rate falls within the experimentally determined range (Goder et al., 2000). Given the highly conserved nature of the translocation machinery in eukaryotes (Cao and Saier, 2003), it is reasonable to assume ER import proceeds at a similar rate in yeast, and thereby permits its use in our calculations. Having now defined both the general protein population and the gateways into the ER lumen, the stage has been set to estimate the flux of protein entering the ER.

## Results

### Estimating the average protein flux into the ER lumen

The time for a specific protein to traverse the ER-membrane depends, in part, on the length of its primary amino acid sequence. In reality, diverse populations of proteins with differing lengths flood the lumenal space. We reason that the abundance-weighted average of amino acids could capture this overall flux. In the simplest case, a single peptide can be envisioned as a mere string consisting of a defined sequence of amino acids. Thus, the total number of amino acids entering the lumen during a given period of time can be captured by calculating the flow of an average length protein (weighted by abundance) into the ER.

**Figure 1:**
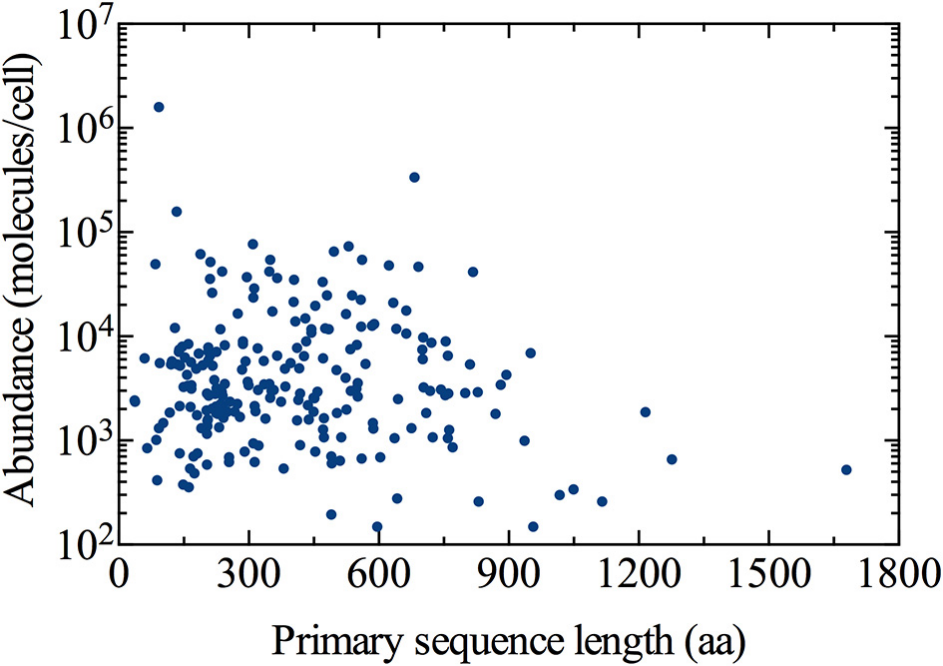
**Distribution of the ER-targeted protein population**. Cellular abundance and subcellular localization data has been obtained from Ghaemmaghami et al. (2003) and Huh et al. (2003), respectively. Primary sequence lengths for all proteins were obtained from the Saccharomyces Genome Database (http://www.yeastgenome.org), accessed June 5, 2014.

We obtained the primary sequence length for each of the 226 ER-targeted proteins quantified by Ghaemmaghami et al. (2003) (Figure 1). Next, the average length of an ER-localized protein was determined by weighting the length of each by its corresponding abundance (number of molecules per cell of a specific protein divided by total number of ER-localized molecules per cell):

(1) $$L=\frac{\sum_{i\;=\;1}^{226}\left(A_{X_{i}}\cdot L_{X_{i}}\right)}{A_{ER}}$$

where *L* is the abundance-weighted average length of an ER-localized protein (in amino acids), *A*_*Xi*_ is the abundance of a specific ER localized protein (in molecules/cell), *L*_*Xi*_ is the length of protein *A*_*Xi*_, and *A*_*ER*_ is the total population of protein localizing to the ER [3,972,824 molecules, determined by analyzing protein abundance data and subcellular localization data presented by Ghaemmaghami et al. (2003) and Huh et al. (2003), respectively].

Using Equation (1), we estimated ~292 amino acids as the abundance-weighted average length of an ER-localized protein, with a minimum length of 36 amino acids corresponding to the OST4 subunit of the oligosaccharyltransferase complex of the ER lumen (ORF: YDL232W), and the NTE1 serine esterase (ORF: YML059C) representing the maximum length of 1679 amino acids. Primary sequence lengths were obtained from the Saccharomyces Genome Database (http://www.yeastgenome.org), accessed June 5, 2014 (Cherry et al., 2012).

Having determined *L*, the average import time (*I*) for a single protein entering the lumen can be calculated using the rate (R) of 8.0 amino acids per second (Goder et al., 2000). Using Equation (2) provided below,

(2) $$I=\frac{L}{R}$$

a value of 36 s is found for *I*. Assuming the number of proteins entering the cell at a given moment in time is proportional to the number of ER translocons present at the surface of the ER membrane, we obtain the following expression that describes the total flux of proteins into the ER:

(3) $$F=\frac{A_{T}}{I}=\frac{A_{T}\;\cdot \;R}{L}$$

where *F* is the flux of proteins entering the ER lumen (in number of molecules per second), *A*_*T*_ is the number of ER translocons per cell (16,500), *I* is the import time (in seconds), and *R* is the translocation rate of 8.0 amino acids per second. A value of ~460 proteins per second is found for *F* when calculated with an *I* of 36 s (see, Table 1).

**Table 1:** **Summary of translocation estimates**.

	**Average**	**Min**	**Max**
*I*	36 s	4.5 s	210 s
*F*	460 molecules/s	78 molecules/s	3700 molecules/s

*The estimated average protein import time (I) and protein flux into the ER lumen (F) have been calculated using Equations (2) and (3), respectively. The abundance-weighted average length of an ER-localized protein (L) of 292 amino acids (aa) and a translocation rate (R) of 8.0 aa/s were used to estimate the averages. In the second and third columns, minimum and maximum translocation estimates have been obtained using Equations (4) and (5), respectively (see, text for details). In the table, s denotes seconds*.

### Estimates for the minimum and maximum protein flux into the ER

The demand for protein folding is highly dynamic, and involves increased flux of specific proteins into the ER that largely depends on the physiological state of the cell. Proteins imported into the ER are highly diverse in many aspects, including primary sequence length (as observed in Figure 1). This implies the number of proteins entering the ER at a given moment in time can vary dramatically.

Accounting for these considerations, we next calculate the range of *F*. This range is fundamentally important because it illustrates the upper and lower theoretical bounds of protein current entering the ER lumen. The absolute minimum flux is defined here as the number of proteins, 1679 amino acids in length, entering the ER per unit time at a translocation rate of 8.0 amino acids per second. On the other hand, we define the absolute maximum flux as the number of 36 amino acid-long proteins entering the ER at a translocation rate of 8.0 amino acids per second. The minimum and maximum import times, *I*_*min*_ and *I*_*max*_, can be calculated using modified forms of Equation (2):

(4) $$F_{\min}=\frac{A_{T}}{I_{\max}},I_{\max}=\frac{L_{\max}}{R}$$

(5) $$F_{\max}=\frac{A_{T}}{I_{\min}},I_{\min}=\frac{L_{\min}}{R}$$

After obtaining values of 210 s for *I*_*max*_ and 4.5 s for *I*_*min*_, we are able to calculate *F*_*min*_ and *F*_*max*_ as 78 and 3700 molecules per second, respectively (Table 1). Interpreting these results in the context of the entire population of 3,972,824 ER-localized proteins [according to Ghaemmaghami et al. (2003), Huh et al. (2003)], this indicates that the ER imports a load of protein between ~0.1 and 5% of its total steady state protein content every minute.

### Estimating the import of a specific protein into the ER

It is often of interest to many researchers modeling various ER processes to determine import rates of specific proteins. This is especially important to those modeling the UPR, as parameters of this nature define the basal inflow of unfolded proteins entering the system, or describe the flux of folding machinery that antagonize stress-elevating phenomena. The above expression for *F* can be extended to obtain such estimates, but must be modified to account for the abundance of the specific protein of interest with respect to the total ER population as a whole. Re-writing Equation (3) we obtain the following expression describing the steady-state flux of a specific protein, denoted *F*_*X*_, into the ER lumen (in molecules per second):

(6) $$F_{X}=\frac{A_{T}\;\cdot \;A_{X}}{I_{X}\;\cdot \;A_{ER}}$$

In this expression, A_*T*_ is the number of ER translocons per cell (16,500), *A*_*X*_ is the abundance of a specific protein X (given in the number of molecules per cell), *I*_*X*_ is the import duration calculated for protein X and *A*_*ER*_ is total population of protein localizing to the ER (3,972,824 molecules).

To illustrate an application of Equation (6), we calculate the flux of the molecular chaperone Kar2p (BiP) into the ER lumen. Kar2p is highly abundant at 337,000 molecules per cell, and has a primary sequence length of 682 amino acids. Substituting its abundance for *A*_*X*_, we calculate *I*_*X*_ as the product of the Kar2p sequence length and the inverse translocation rate (*I*_*X*_ = 85 s when calculated for Kar2p with an average translocation rate of 8.0 amino acids per second). Doing so, we determine the flux of Kar2p into the ER to be 16,466 molecules per second.

## Discussion

**Figure 2:**
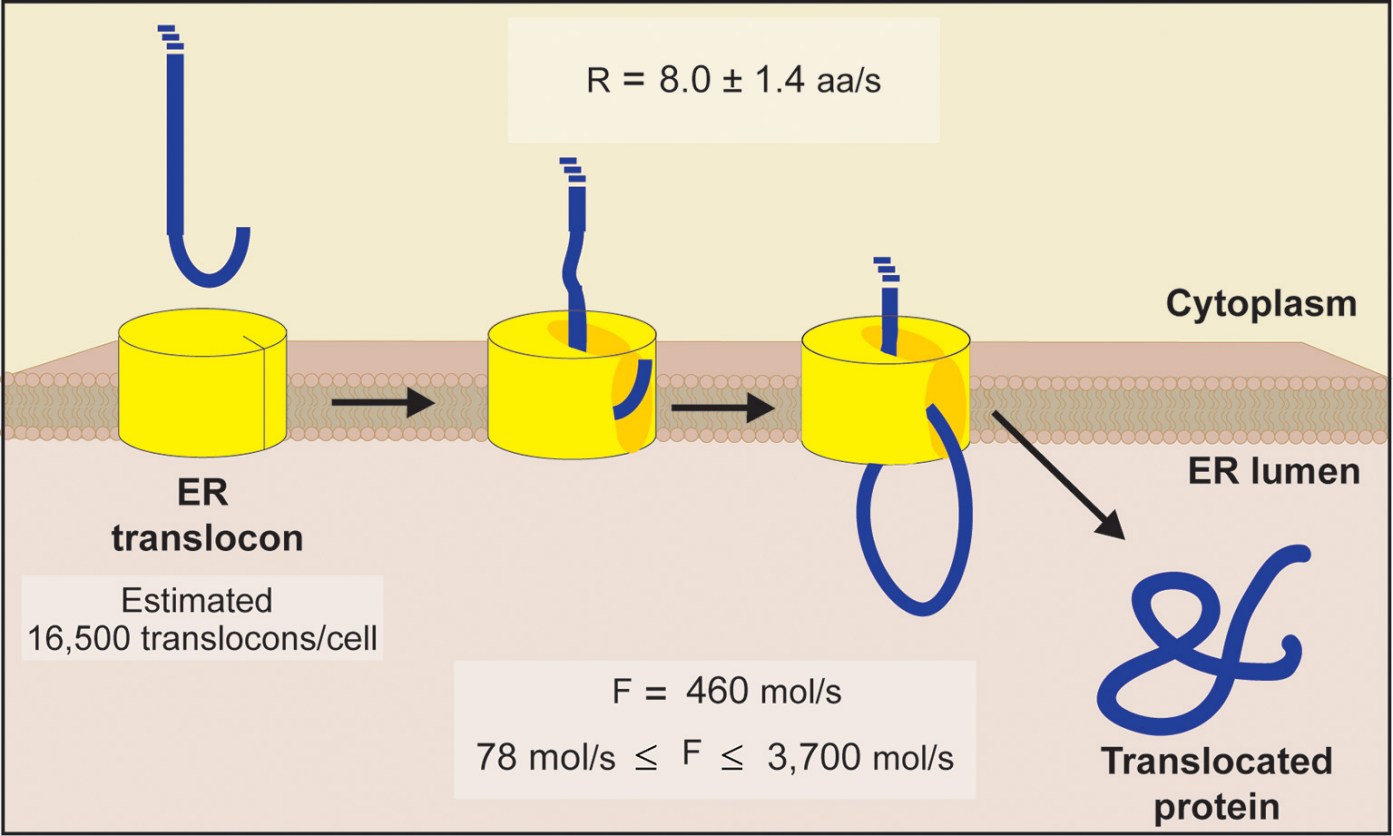
**Schematic diagram of ER translocation summarizing our protein flux estimations**. An estimate of 16,500 translocons per cell was obtained by comparing the abundance of each essential subunit comprising the yeast ER translocon (Sec61p, Sec62p, Sec63p, Sss1p, and Kar2p). This value matches the abundance of Sec62p, the limiting subunit inferred from proteomic information (Ghaemmaghami et al., 2003). Using translocation rates determined in a eukaryotic system (Goder et al., 2000), we next estimated the ER to experience an average flux of 460 molecules/s, with an absolute minimum and maximum flux of 78 molecules/s and 3700 molecules/s, respectively [see Equations (1–5) for details].

We set out to provide data driven estimates for total protein flux into the ER. An illustration summarizing our estimations is presented in Figure 2. After first estimating the number of ER translocons present in a single cell, kinetic parameters determined in a eukaryotic system were used to define the rate of translocation of proteins entering the ER lumen. Subsequently, we estimated the ER to experience an average protein inflow of 460 proteins per second. With this value representing the import of an average length protein (weighted by abundance), it accounts for the total amino acids entering the lumen and therefore respects the diversity of proteins associated with this organelle. Even in light of these considerations, we do not account for the time delay between protein import events, nor do we account for other physicochemical influences (aside from primary sequence length) that could impact this event as well.

Length variations likely hold tremendous influence over the number of distinct peptides entering the lumen in a given period of time. To characterize the effect that protein length has on total flux into the ER, we determined the absolute lower and upper bounds for protein import into the ER, based on the maximal and minimal lengths of all ER-targeted proteins, respectively. Provided this absolute range corresponds to between ~0.1 and 5% of protein content within the ER at steady state, our calculation implies that the combined effort of export and degradation machinery must dismiss roughly 3973–198,641 proteins every minute to maintain protein homeostasis in yeast.

We believe our estimates could be of great value to investigators constructing models of ER processes. The equations presented here can be used to estimate source terms for both specific proteins and larger protein populations entering the ER. Interestingly, it appears that our estimates regarding total protein flux in the ER lumen align well with a corresponding parameter value used in a recent model of the yeast unfolded protein response. Pincus et al. (2010) used parameter-fitting techniques to define the flux of unfolded protein into the ER as 310 proteins per second. This value lies within our absolute range of 78–3700 molecules per second (Table 1). While 310 molecules per second is in the lower end of our range, this value could be more appropriate for modeling the UPR as decreased protein translocation is thought to be one of the consequences of UPR activation (due to the challenge ER stress imposes on the chaperone population). Nevertheless, the estimates presented here may improve the biological accuracy of ER models in the near future.

To our knowledge, our estimate concerning the number of ER translocons per cell is the first that considers its composition in the context of the cellular abundance of each of its core subunits and auxiliary components. We used the abundance of molecules encoded by essential genes to define the translocon population. Interestingly, our estimate of 16,500 translocons per cell excludes the Sbh1p subunit, which is limited to 217 molecules per cell (Ghaemmaghami et al., 2003). Admittedly, yeast mutants lacking Sbh1p are viable, with intact, although impaired, protein translocation into the ER (Finke et al., 1996). This suggests a biologically important role for Sbh1p in the translocon assembly, which could involve aiding the import of a specific subset of proteins into the ER, or improving the overall efficiency of ER translocation.

We acknowledge our estimates were made possible by oversimplifying the process of translocation. For simplicity, we only considered unidirectional protein flow into the ER. Furthermore, we did not account for specific cotranslational and posttranslational translocation considerations, nor did we consider the cycling between ribosome-bound and ribosome-free states. The precise stoichiometry of targeting and auxiliary components distinguishing ER translocons operating in cotranslational vs. posttranslational processes is needed to further distribute our estimated 16,500 ER translocons between each. Also requiring further distribution is the overall population of protein localizing to the ER. If the overall translocon population were split into two subgroups, a consistent methodology would entail each distinct ER-localizing species to be divided among those imported cotranslationally vs. those imported after translation. Indeed, an additional layer of complexity would be provided if yet a third subpopulation were defined as well, composed of proteins that traverse the ER membrane via either mechanism as described by Ng et al. (1996).

Dividing flux estimates between co- and posttranslational translocation mechanisms is further hindered by process-specific details. This is especially true for the former process, which is dependent on the binding of an SRP to an SRP-receptor. The rate of translation carried out by ribosomes docked to the translocon also impacts this process. Kinetic rates for translation and SRP-targeting have been determined experimentally in mammalian systems (Hershey, 1991; Goder et al., 2000). We are unaware of corresponding parameters in yeast. Nevertheless, interactions between the pool of protein awaiting entry into the ER, SRP (and the SRP receptor), ribosomes, and the ER translocon are highly dynamic in nature. Stochastic models would be better suited for adequately addressing these considerations in the future.

Although our estimates are theoretical, we believe they offer valuable insight regarding the flow of protein entering the ER lumen. Taken together with existing proteomic information, we intend the equations contained herein to provide quantitative biologists investigating ER processes with a tool for estimating the import of any ER localizing protein in yeast. It is important that the modeling community continues to provide resources to aid in the identification of realistic parameters, as the use of inaccurate or biologically irrelevant parameter values can jeopardize the reliability of model predictions. It should also be well understood that reliable parameter estimates are crucial for gaining insights from models in systems and computational biology, especially those involving non-linear phenomena. Nevertheless, this work merely represents an initial step toward quantifying the flow of protein entering the ER lumen. More accurate characterization of ER protein flux awaits further experimental investigation. With the ongoing development of critical biotechnologies, such as nanosensors and novel fluorescent markers, objective measurements of ER protein import may not be far away.

### Conflict of interest statement

The authors declare that the research was conducted in the absence of any commercial or financial relationships that could be construed as a potential conflict of interest.
